# Clinical ethics recommendations for the allocation of intensive care treatments in exceptional, resource-limited circumstances: the Italian perspective during the COVID-19 epidemic

**DOI:** 10.1186/s13054-020-02891-w

**Published:** 2020-04-22

**Authors:** Marco Vergano, Guido Bertolini, Alberto Giannini, Giuseppe R. Gristina, Sergio Livigni, Giovanni Mistraletti, Luigi Riccioni, Flavia Petrini

**Affiliations:** 1 Società Italiana di Anestesia Analgesia Rianimazione e Terapia Intensiva, Viale dell’Università 11, 00185 Rome, Italy; 2 Laboratorio di Epidemiologia Clinica, Dipartimento di Salute Pubblica, Istituto di Ricerche Farmacologiche Mario Negri IRCCS, Milan, Italy

## Background

On February 21, 2020, the first person-to-person transmission of severe acute respiratory syndrome coronavirus 2 (SARS-CoV2), the virus causing coronavirus disease 2019 (COVID-19), was identified in Italy. In the following days, despite the restrictive public health measures applied to avoid the spread of the infection [1], the number of cases sharply increased. As of March 8, 2020, Italy was the 2nd most affected country in the world.

In one of the largest reports from China, 5% of COVID-19 patients required admission to the intensive care unit (ICU) [2]. Since the beginning of the COVID-19 outbreak, the availability of ICU beds has been recognized as one of the major public health concerns in Italy, where a total of 5090 ICU beds (8.42/100,000 inhabitants) were reported in 2017 [3]. Despite further efforts have been done to contain the number of cases and extraordinary measures have been put in place, the dramatic increase of ICU admission abruptly overwhelmed the ICU capacity, mostly in Lombardy and in the nearby regions of Northern Italy.

From the evidence available so far, a considerable proportion of subjects diagnosed with COVID-19 infection requires ventilatory support due to severe hypoxemia in the context of interstitial pneumonia. The interstitial lung disease is potentially reversible, but the acute course of the disease can last several days, and ventilatory support may be needed for weeks [4]. These clinical considerations imply that caring for patients with severe pneumonia from COVID-19 can be very demanding in terms of the number of devices and staff required.

As of March 6, 2020, the Italian Society of Anesthesia, Analgesia, Resuscitation and Intensive Care (SIAARTI) issued a series of recommendations [5] and relevant ethical considerations to better inform the clinicians involved in the care of critically-ill COVID-19 patients, in a setting where a disproportionate number of patients requiring life-sustaining treatments was rapidly saturating both the existing and the newly set-up ICU beds. The most relevant recommendations are summarized in Table 1.

**Table 1:** Summary of the ethical recommendations issued by the Italian Society of Anesthesia, Analgesia, Resuscitation and Intensive Care (SIAARTI) during the COVID-19 epidemic

**Allocation of ICU resources**	Allocation of ICU resources is a complex and delicate task. Criteria for ICU admission and discharge under exceptional, resource-limited circumstances must be flexible and should be locally adapted according to the availability of resources, the potential for inter-hospital patient transfer, and the ongoing or foreseen number of hospital and ICU admissions. These criteria apply to every patient potentially in need of ICU admission, not only to COVID-19 infected patients.
**Triage principles and criteria**	Age, comorbidities, and the functional status of any critically ill patient should carefully be evaluated. A longer and, hence, more “resource-consuming” clinical course may be anticipated in frail elderly patients with severe comorbidities, as compared to a relatively shorter and potentially more benign course in healthy young subjects. The underlying principle would be to save limited resources which may become extremely scarce for those who have a much greater probability of survival and life expectancy, in order to maximize the benefits for the largest number of people. In the worst-case scenario of complete saturation of ICU resources, a “first come, first served” criterion is not recommended, as it would ultimately result in denying access to ICU care to a large number of potentially curable patients.
**Advance healthcare directives**	The presence of advance healthcare directives or advance care planning should be carefully evaluated, especially for patients affected by severe chronic illnesses. These plans should be shared as much as possible between the patient, their proxies, and all the healthcare staff involved in patient care. A decision to deny admission to the ICU by applying a “ceiling of care” should always be motivated, communicated, and documented. The decision to withhold invasive mechanical ventilation does not necessarily imply that other, non-invasive, modalities of ventilatory support should also be withheld.
**Decision-making process**	The decision to withhold or withdraw life-sustaining treatments must always be discussed and shared among the healthcare staff and, when possible, the patients and/or their proxies. A second opinion (e.g., from Regional Healthcare Coordination Centres, or from other recognized or designated experts) may be useful when dealing with particularly difficult or distressing cases.
**Palliative care**	Appropriate palliative care must always be provided to hypoxemic patients when a decision to withhold or withdraw life-sustaining treatments is made. Palliative care should be provided according to national or international recommendations, as a matter of good clinical practice.
**ICU trials and proportionality of care**	Every admission to the ICU should be considered and communicated as an “ICU trial.” The appropriateness of life-sustaining treatments should be re-evaluated daily, considering the patient’s history, current clinical course, wishes, expected goals, and proportionality of ICU care. When a patient is not responding to prolonged life-sustaining treatments, or severe clinical complications arise, a decision to withhold or withdraw further or ongoing therapies should not be postponed in a resource-limited setting during an epidemic.
**Networking and family care**	Networking among healthcare professionals is essential to share clinical expertise. Dedicated time and resources should be anticipated for team debriefing and monitoring of burnout symptoms or moral distress among the healthcare staff once time permits. Also, the impact of restricted visiting policies on families and proxies should be considered, especially when the death of a loved one occurs during times of complete restriction of family visits.

## General principles

The emerging epidemic is leading to a substantial increase in the number of patients requiring prolonged ventilatory support for acute respiratory failure, potentially resulting in severe imbalances between the population clinical needs and the overall availability of ICU resources. In this scenario, criteria for ICU admission (and discharge) may need to be driven not only by the principles of clinical appropriateness and proportionality of care, but also by criteria of distributive justice and appropriate allocation of the healthcare resources, that may be more limited than usual.

The primary aim of these recommendations is therefore to supply a common framework for the admission of patients to intensive care treatments in resource-limited circumstances. These recommendations should be shared maximally within all the involved healthcare providers.

Bioethical reasoning has inspired several operative instructions for the field of disaster medicine. In this area, healthcare providers must be supported during their difficult decision-making process. As an extension of the principle of proportionality of care, in the context of a severe shortage of ICU resources, these should be preferentially allocated to patients with the higher possibility of therapeutic success. Therefore, the aim is to privilege the greater chance to successfully overcome critical illness with a greater probability to maintain a good quality of life.

A single patient’s actual need for ICU treatments should be therefore integrated with additional criteria for ICU admission, taking into account the type and severity of the current disease, comorbidity, the presence and reversibility of organ failures, and the potential for recovery. It follows that within the foregoing context, the “first come, first served” criterion for ICU admission does not necessarily have to be followed.

Because of the rare occurrence of large-scale catastrophic events, the healthcare staff may not be very familiar with the criteria applied for triage during mass casualty events. The availability of resources may not always be part of the clinical decision-making process operated on a single patient, until resources become so limited that it is not possible to treat all patients who may hypothetically benefit from a specific treatment.

The application of restrictive (rationing) policies is justifiable only if all the relevant stakeholders (“task forces,” hospitals, institutions) have already tried to increase the availability of resources and have already assessed the feasibility and safety to transfer patients to other hospitals.

As previously mentioned, a change in the ICU admission policy should be shared maximally among the staff involved. Moreover, the patients who are affected by the application of new, more stringent criteria of eligibility for ICU admission (and/or their proxies) should be informed of the extraordinary nature of the measures in place, as a matter of duty of transparency and to maintain confidence in the health service.

An additional aim of the recommendations is to support the clinicians when dealing with individual patients, as hard and complex decisions may be ethically and emotionally demanding.

## Data Availability

Not applicable.
